# Genome Sequences of Seven *Megrivirus* Strains from Chickens in The Netherlands

**DOI:** 10.1128/MRA.01207-20

**Published:** 2020-11-19

**Authors:** Kirsty T. T. Kwok, Myrna M. T. de Rooij, Aniek B. Messink, Inge M. Wouters, Marion P. G. Koopmans, My V. T. Phan

**Affiliations:** a Department of Viroscience, Erasmus MC, Rotterdam, The Netherlands; b Institute for Risk Assessment Sciences (IRAS), Utrecht University, Utrecht, The Netherlands; DOE Joint Genome Institute

## Abstract

We report seven chicken megrivirus genome sequences identified in chicken fecal samples from a broiler farm in The Netherlands. The sequences were determined using metagenomic sequencing and would expand our understanding of the genome diversity of megriviruses.

## Abstract (precis)

We report seven chicken megrivirus genome sequences identified in chicken fecal samples from a broiler farm in the Netherlands. The sequences were determined using metagenomic sequencing and would expand our understanding of the genome diversity of megriviruses.

## ANNOUNCEMENT

Viruses in the *Picornaviridae* family possess a positive-sense single-stranded RNA genome and cause a broad range of diseases in humans and animals (1). Among the 63 genera in this family, at least 15 genera, including *Megrivirus*, have been identified from avian sources (2). Megriviruses have been detected in both healthy and diseased poultry (3, 4) and are suspected to cause transmissible viral proventriculitis in chickens (5) and hepatitis in turkeys (6). Here, we report 7 nearly complete megrivirus genome sequences identified from 7 out of 8 pooled chicken fecal samples that we sequenced as part of a virome profiling study of farm animals. The samples consisted of pooled fresh fecal droppings from 3 to 4 chicks (Gallus gallus domesticus), collected in the same flock during weeks 4 and 5 of the production cycle from a broiler farm in June 2019 in The Netherlands (Table 1). No clinical signs in the examined flock were reported at the time of sampling.

**TABLE 1:** Sequence data for 7 megrivirus strains

Sample identifier	Production wk no.	Total no. of reads	No. of megrivirus-specific reads	Depth of coverage (×)	Sequence length (nt)	G+C content (%)	GenBank accession no.	SRA accession no.
V_M_013	4	2,595,998	38,347	576	9,590	45.4	MW054505	SRX9349804
V_M_014	4	4,046,054	2,292	33	9,410	45.1	MW054506	SRX9349805
V_M_015	4	4,765,832	33,371	446	9,566	45.3	MW054507	SRX9349806
V_M_016	4	4,172,232	23,041	307	9,592	45.4	MW054508	SRX9349807
V_M_017	5	2,020,090	4,993	86	9,561	45.2	MW054509	SRX9349808
V_M_018	5	3,901,518	2,401	34	8,993	45.4	MW054510	SRX9349809
V_M_019	5	4,286,694	6,302	88	9,294	45.2	MW054511	SRX9349810

The genome sequences of the virus were generated using metagenomic sequencing. The fecal suspension (30% [wt/vol] in phosphate-buffered saline) was centrifuged for 10 min at 10,000 × *g*. The supernatant was collected and treated with TURBO DNase (Invitrogen). Virion-protected nucleic acid was extracted using the QIAamp viral RNA minikit (Qiagen). Reverse transcription was performed using nonribosomal random hexamers (7) and SuperScript III reverse transcriptase (Invitrogen), followed by second-strand cDNA synthesis using Klenow fragment 3′–5′ exo- (New England BioLabs). The resulting DNA was subjected to library preparation using a Nextera XT DNA library preparation kit (Illumina) following the manufacturer’s instructions. The library was then purified and size selected using AMPure XP magnetic beads (Beckman Coulter). The final library was sequenced in paired-end format on the Illumina MiSeq platform using reagent kit v3 (600 cycles; Illumina). The total number of reads generated per sample ranged between 2,020,090 and 4,765,832. The raw reads were analyzed using the automated pipeline Genome Detective Virus Tool v1.126 (8), which utilizes Trimmomatic (9) for adapter and quality trimming, DIAMOND (10) for viral read identification, and metaSPAdes (11) for *de novo* assembly of the sorted viral reads. *De novo*-assembled genome sequences were inspected and annotated using Geneious v2020.2.3 (12). The lengths of the 7 nearly complete megrivirus genomes range from 8,993 to 9,592 nucleotides (nt). The depths of coverage range from 34× to 576×. According to BLAST searches, these strains shared 83% to 84% identity at the nucleotide level with chicken picornavirus 5 isolate 27C from Hong Kong (2008; GenBank accession number KF979336), chicken megrivirus strain MG9567 from Brazil (2012; MH806866), and *Picornaviridae* sp. isolates w3chi090pic1 and w3chi091pic1 from China (2018; MT138368 and MT138369, respectively). These reported genome sequences shared 92% to 93% identity at the amino acid (aa) level when comparing viral polyprotein amino acid sequences. The pairwise nucleotide difference of the complete coding region among the 7 strains ranges from 2 to 18 nt; the pairwise amino acid difference ranges from 0 to 4 aa.

To conclude, we report 7 megrivirus genome sequences identified in The Netherlands. The prevalence of these viruses might have been overlooked in the poultry population thus far. Future studies should investigate the prevalence and diversity of megriviruses and their potential clinical implications.

### Data availability.

The genome sequences described in this study have been deposited in GenBank under the accession numbers MW054505 to MW054511. The raw reads are available in the SRA under the BioProject accession number PRJNA670873.
